# Protein expression and transcription profiles of three strains of *Aeromonas salmonicida* ssp. salmonicida under normal and iron-limited culture conditions

**DOI:** 10.1186/1477-5956-12-29

**Published:** 2014-05-19

**Authors:** Simon Menanteau-Ledouble, Julia Kattlun, Katharina Nöbauer, Mansour El-Matbouli

**Affiliations:** 1 Clinical Division of Fish Medicine, University of Veterinary Medicine, Veterinärplatz 1, Vienna 1210, Austria; 2 VetCore Facility for Research, University of Veterinary Medicine, Vienna, Austria

**Keywords:** Fish, Virulence, ATP-synthetase F, Alkyl hydroperoxide Reductase, Histone-like nucleoid structuring protein, Enolase, hns, RT-qPCR, Iron-regulated proteins

## Abstract

**Background:**

*Aeromonas salmonicida* is an important fish pathogen that produces a wide and varied array of virulence factors. Here we used iron deprivation by addition of the chelator 2’2-dipyridyl to induce the expression of several such virulence factors in three isolates of *Aeromonas salmonicida* (one avirulent and two virulent). By using SDS-PAGE followed by mass spectrometry, we identified proteins that appeared differentially expressed under these conditions. The differential transcription of the identified gene products were subsequently measured by reverse transcription quantitative real-time PCR (RT-qPCR).

**Results:**

Our initial screening using SDS-PAGE identified five proteins that appeared differentially expressed in virulent and avirulent isolates or, within the same isolates, between bacteria cultivated under iron-rich or iron-deprived conditions. The transcription of the genes coding for these proteins were subsequently quantified by RT-qPCR. Results of this analysis demonstrated that the gene coding for alkyl hydroperoxide reductase (AhpC), a protein involved in oxidative stress response, was transcribed at a higher rate in the virulent strain as compared to the avirulent strain. Additionally, it was observed that addition of an iron chelator to the culture medium lead to a reduction of the transcription levels of the regulatory histone-like nucleoid structuring protein (H-NS). This was consistent in all three isolates. On the other hand, the transcription levels of the virulence array protein (VapA) and the protein ATP-synthetase F (ATPF) displayed only limited changes, despite being the dominant component of a protein fraction that displayed changes during the preliminary SDS-PAGE screening. This was true regardless of the culture conditions and of the isolates considered. Finally, transcription of the enzyme enolase was upregulated in the iron-deprived broths in all isolates.

**Conclusions:**

We identified several genes differentially expressed under culture conditions known to lead to the overexpression of virulence factors. In addition, we identified alkyl hydroperoxide as being overexpressed in the virulent isolates compared to the avirulent isolates. The results from this study will contribute to enhance our understanding of the virulence of *A. salmonicida* and may suggest new directions for further research.

## Background

First described in the 19th century, *Aeromonas salmonicida* is considered today as one of the oldest known fish pathogens. Much has since been discovered with regard to its virulence including identification of extracellular protein. For example, the P1 and P2 proteases, caseinase and metallo-caseinase [1] and glycerophospholipid cholesterol acyltransferase (GCAT) [2]. However, the importance of such extra-cellular products was shown to be limited prompting some investigators to suggest that bacterial virulence may predominantly be attributed to bacterial growth [3]. A number of adhesion factors such as the A-layer, type I and type IV pili have also been identified [4-6]. More recently, the presence of a type III secretory apparatus [7] was also described. Unlike other virulence factors, the T3SS of *A. salmonicida* has been reported to be playing a major role in bacterial virulence [8-10]. The T3SS system is remarkable because of its thermo-sensitivity, which is lost when the organism is cultured at temperatures above 25°C [11,12]. Such thermo-sensitivity is of particular interest because the bacterium can be cultured at temperatures as high as 34.5°C, with a recommended temperature for the culture of Aeromonads as high as 28°C [13]. Therefore, it is likely that several clinical isolates have lost the type III secretion system they once carried [12] and have become avirulent. This loss may have occurred for the type strain of *A. salmonicida* ssp. *salmonicida* ATCC 33658 T, which is avirulent and does not possess a type III secretion system [8,14].

In most bacterial pathogens, virulence factors are tightly regulated [15]. One such regulator is the ferric uptake regulator (Fur) which is sensitive to the oxidative-stress response [16]. Fur controls bacterial response to oxidative molecules, including those generated by free radicals from iron, as well as the acquisition of iron [17]. In addition, Fur and related metalloregulatory proteins are also involved in the regulation of a number of virulence genes [16] in a variety of organisms, including *E. coli*[18], *Streptococcus*[19], *Pseudomonas*[20], *Shigella*[21], *Edwardsiella*[22] and *Vibrio*[23,24]. Not surprisingly, such regulatory mechanisms of bacterial virulence are present in *A. salmonicida*. For example, molecules associated with the quorum sensing system have been described [25,26] and shown to play a role in regulating the production of proteases [26] including Asap1 [27]. Similarly, a correlation was demonstrated between iron depletion and the production of virulence factors such as superoxide-dismutase [28], hemin receptor HutE, and the expression levels of genes encoding a putative DapD and Flp/Fap-pilin proteins [29]. Finally, three iron-regulated outer membrane proteins (IROMPs) have been described in *A. salmonicida*[30], all were TonB dependent: The first IROMP was a ferric siderophore receptor of 85 kDa in size while the second was identified as a colicin receptor homologue (FstC) of 73 kDa and likely to also act as a ferric receptor homologue. Finally, the last protein was 76 kDa in size and homologous to the heme receptor protein HutA from *Vibrio cholera*.

In *A. salmonicida*, it has been shown that the bacterial growth was slowed in iron-deprived cultures [30]. This is especially important during the infectious process because free iron is kept at a minimal level inside the host [31]. In fact, it has been shown that activation of human monocytes by the interferon-γ leads to reduction in their uptake of transferrin, therefore reducing the availability of intracellular iron [31]. For this reason, therapeutants aiming at further reducing the iron availability and acquisition for the bacterial pathogen have received scrutiny as alternative to antibiotherapy: Adjunction of the iron chelator deferasirox was shown to complement the efficiency of vancomycin against methicillin-resistant *Staphylococcus aureus*[32]. On the contrary, deferoxamine, another chelator, had a negative effect on the outcome of infection by *Yersinia* but only a limited effect on *Klebsiella* infection and no noticeable effect on infections with *Aeromonas hydrophila*[33]. Furthermore, inhibitors of siderophore production such as acyl-sulmamoyl adenosines have also been tested and proved efficacious against *Mycobacterium tuberculosis* and *Yersinia pestis*[34]. Finally, the monoterpenoid phenol carvacrol was shown to suppress the virulence of *Salmonella enterica*[35]. Unfortunately, despite these promising findings, this approach has yet, to the best of our knowledge, to be applied against fish pathogens. It is worth noting, however, that carvacrol is present in essential oils and might contribute to the protective effect of these when administrated as food feed additive [36,37].

The importance of iron availability in virulence and its regulatory mechanisms is therefore well established. Indeed, it was discovered that, at least as far as the major IROMPS were concerned, culture under iron-limited conditions up-regulated the same proteins than growth *in vivo* during the infectious process [30]. Consequently, the comparison of the transcription profile of an isolate between iron-rich and iron-deprived conditions as well as between virulent and avirulent isolate is likely to facilitate the identification of factors uniquely associated with a virulent phenotype. Here, we applied a preliminary SDS-PAGE screening, followed by quantitative RT-PCR to compare the protein transcription and expression profiles between three different isolates: the avirulent type strain ATCC 33658 T as well as two clinical isolates A-14390 and A-15233 under normal and iron-deprived culture conditions. Results from this study will enhance our understanding of the virulence in aquatic pathogens such as in *A. salmonicida*.

## Results

### Preliminary SDS-PAGE screening

**Figure 1:**
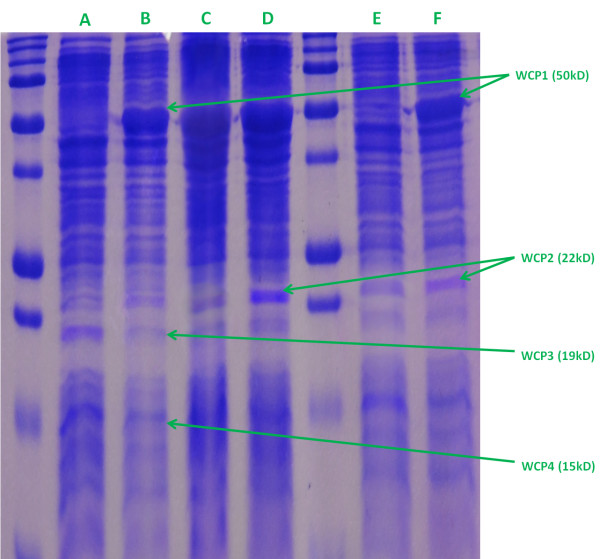
**SDS**-**PAGE of whole**-**cell preparations**, **showing the 4 significant bands that were purified and analyzed.** The wells were loaded as follow: **A**- Isolate ATCC 33658 T (avirulent) cultivated under normal conditions; **B**- Isolate ATCC 33658 T (avirulent) cultivated under iron-deprived conditions; **C**- Isolate A-14390 (virulent) cultivated under normal conditions; **D**- Isolate A-14390 (virulent) cultivated under iron-deprived conditions; **E**- Isolate A-15233 (virulent) cultivated under normal conditions; **F**- Isolate A-15233 (virulent) cultivated under iron-deprived conditions.

When examined by SDS-PAGE, the various samples produced similar pattern of bands (cf. Figure 1). However, five protein bands ranging from 50 kD to 15 kD in size appeared differentially expressed. Those five bands were excised, analysed by mass spectrometry and identified based on their molecular weight and the analysis of their mass spectra by Mascot.

A band of approximately 50 KD (WCP1) was observed in the whole cell crude extract and appeared more strongly visible under iron-limited conditions in the ATCC 33658 T and A-15233 isolates. Among the proteins identified in this fraction (Table 1), the protein with the highest score was the virulence array protein A (VapA; NCBI reference sequence: [Genbank: CAG70991.1] score 5036.2). The enzyme enolase ([Genbank: YP_001143191.1]; 1752) and a LamB like protein ([Genbank: YP_001142909.1]; score of 856) were also present with a high score.

**Table 1:** Summary of the main proteins investigated during this study

**Genbank reference number of identified proteins**	**Predicted function**	**Behavior**
**Band WCP 1 (50 kD)**		
[CAG70991.1]	Virulence array protein (part of the crystalline layer)	Overexpressed under iron-deprived conditions in ATCC
[YP_001143191.1]	Enolase (metalloprotease; plasminogen binding)	33658 T and A-15233.
**Band WCP 2 (22 kD)**		Overexpressed in the virulent strains A-14390 and A-15233 under iron-deprived conditions.
[YP_001142399.1]	Alkyl Hydroperoxide Reductase C (Resistance to oxidative stress)	
[YP_001142899.1]	Frr (Ribosome recycling factor)	
**Band WCP 3 (19 kD)**		Under-expressed under iron-deprived conditions in ATCC 33658 T.
[YP_001144017.1]	AtpF gene product (resistance against acidic conditions)	
**Band WCP 4 (15 kD)**		Under-expressed under iron-deprived conditions in ATCC 33658 T.
[YP_001142759.1]	H-NS (Transcriptional regulator; inhibitor of several virulence genes)	
**Band OMP 2 (50 kD)**		Overexpressed under iron-deprived conditions in A-15233.
[YP_001143191.1]	Virulence array protein (part of the crystalline layer)	
[YP_001143191.1]	Enolase (metalloprotease; plasminogen binding)	

Similarly, a band of 22 kD (WCP2) appeared to be stronger, under iron-deprived conditions, in the virulent isolates. Two proteins dominated this fraction: an alkyl hydroperoxide reductase gene product (AhpC; [Genbank: YP_001142399.1]; score 647) and the ribosome recycling factor (*frr* gene product; [Genbank: YP_001142899.1]; 472).

Two bands of 19 kD (WCP3) and 15 kD (WCP4) were observed in the whole cell crude preparations. In the reference strain ATCC 33658 T, these bands displayed low intensity under iron-limited conditions while the expression level did not display any obvious change in the virulent isolates. The 19 kD band contained mostly ribosomal proteins with the highest scores from RplF ([Genbank: YP_001143761]; score 783), RplE ([Genbank: YP_001143764.1]; 695) alongside AtpF ([Genbank: YP_001144017.1]; score of 550).

The 15 kD band contained ribosomal protein RplL ([Genbank: YP_001140619.1]; score of 416) and the regulatory protein H-NS ([Genbank: YP_001142759.1]; score of 395).

Among the outer-membrane proteins only one band of 50 kD (OMP2) was over-expressed (cf. Additional file 1: Figure S1) in isolate A-15233 under iron-limited conditions. This band mostly comprised the proteins VapA (score 3930) and Enolase (score 232).

### Real-time qPCR

#### Comparison between virulent and avirulent strains

**Figure 2:**
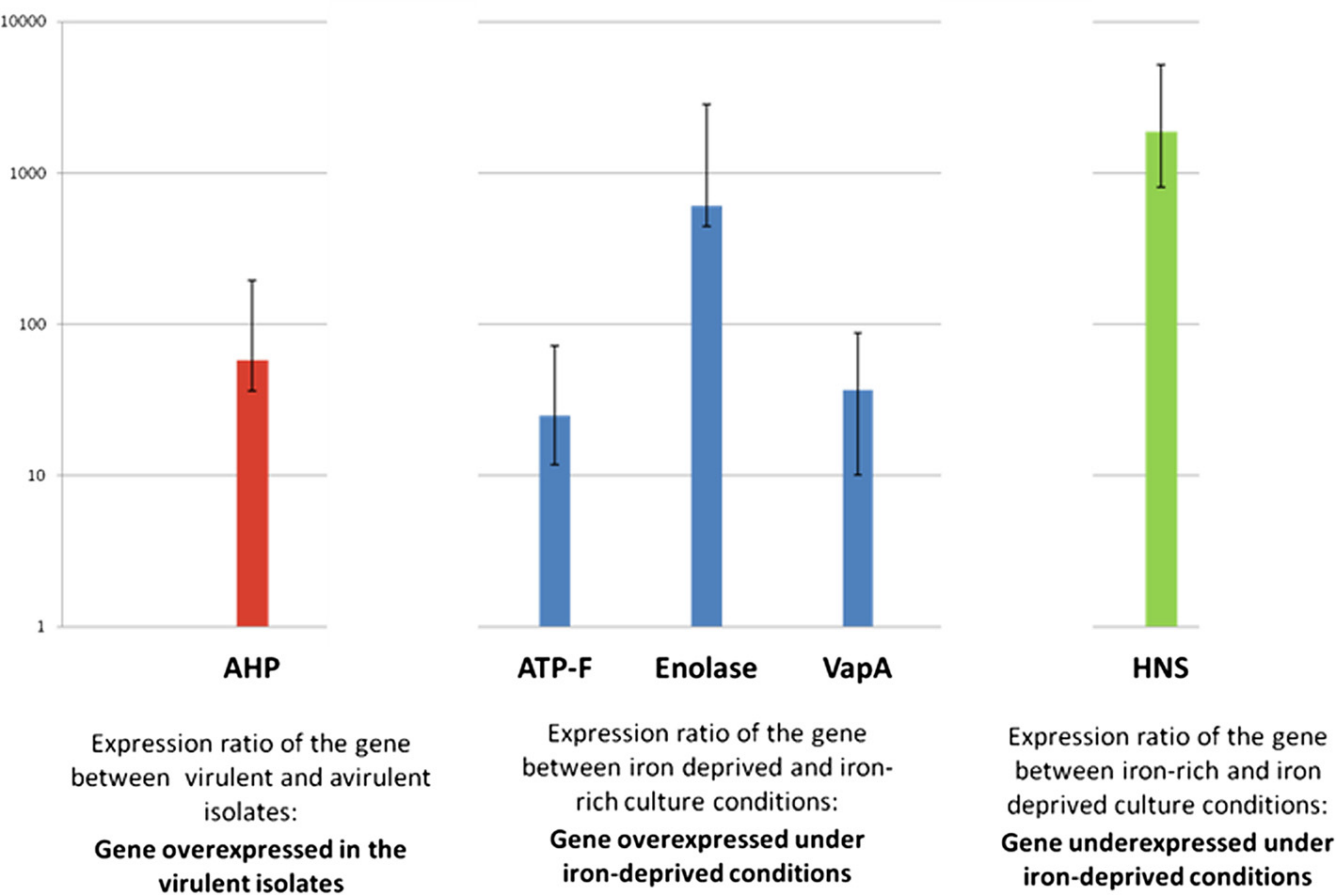
**Expression Ratios of the various genes investigated by RT**-**qPCR during this study.**

RT-qPCR was performed on genes selected based on the results from the SDS-PAGE screening and its results are summarised in Figure 2 and Table 2. Only one gene, *ahpC*, was transcribed at a significantly higher rate in the virulent isolates compared to the avirulent ATCC 33658 T strain (mean fold change: 5.8E^+01^ ± 1.86E^+00^, as calculated with the 2^-ΔΔC^_T_ method). This higher rate of transcription was true under both iron rich and iron-deprived culture conditions.

**Table 2:** **Summary of the results from the RT**-**PCR analysis**

		**Mean fold change**	**Upper limit C.I**	**Lower limit C.I**
AHPC	Overexpressed in virulent strains compared to the avirulent ATCC 33658 T			
	Transcription Ratio of AHPC in the virulent strains compared to ATCC 33658 T:	5.80E + 01	1.38E + 02	2.17E + 01
ATP-F	Overexpressed under iron-deprived conditions			
	Transcription Ratio of the iron-poor cultures compared to the iron-rich ones:	2.50E + 01	4.73E + 01	1.32E + 01
Enolase	Overexpressed under iron-deprived conditions			
	Transcription Ratio of the iron-poor cultures compared to the iron-rich ones:	6.10E + 02	2.23E + 03	1.68E + 02
H-NS	Under-expressed under iron-deprived conditions			
	Transcription Ratio of the iron-rich cultures compared to the iron-poor ones:	1.89E + 03	3.29E + 03	1.09E + 03
VapA	Overexpressed under iron-deprived conditions			
	Transcription Ratio of the iron-poor cultures compared to the iron-rich ones:	3.65E + 01	5.14E + 01	2.64E + 01

#### Comparison between iron-rich and iron deprived conditions

WCP1 and OMP2 (50 kD), both contained the same two proteins of interest: VapA and enolase. RT-qPCR failed to detect any significant difference in the level of transcription of VapA (mean fold change: 3.64E + 01, with a confidence interval ranging from 2.64E + 01 to 5.14E + 01). Enolase, on the other hand was more clearly upregulated (mean fold change: 6.10E + 02, ranging from 1.68E + 02 to 2.23E + 03).

The 22 kD band contained AhpC, a protein that was found to be upregulated in the virulent isolates. Additionally, it was found that deprivation of iron also led to a slightly elevated transcription of this gene in all strains (mean fold change: 3.14E^+01^).

The 19 kD band contained AtpF and, when examined by SDS-PAGE, it appeared fainter in the ATCC 33658 T isolate when cultivated under iron-deprived conditions. This suggested that the protein might be less expressed under these conditions. However, analysis by qPCR indicated a minor up-regulation at the level of transcription (2.50E + 01, ranging from 1.32E + 01 and 4.73E + 01).

The 15 kD band contained the regulator H-NS. Results of the RT-qPCR revealed that it was significantly overexpressed under iron-replete conditions (average transcription ratios of 1.89E + 03, ranging from 1.09E + 03 to 3.29E + 03).

## Discussion

In order to investigate the virulence factors of the fish pathogen *A. salmonicida*, three bacterial isolates, one known to be avirulent and two virulent ones were cultivated under iron rich and iron deprived conditions. The bacteria in these cultures were lysed and the cell lysates were analysed on SDS page. Five fractions (of size ranging from 50 to 15 kD) that appeared differentially expressed between the virulent and avirulent isolates or between iron-rich and iron-deprived culture conditions were excised and analysed by mass-spectrometry and identified by Mascot.

Fraction WCP1 contained proteins of ~50 kD in size. These were found to be upregulated under iron-deprived conditions upon examination of the SDS-PAGE. Based on the PMSS method the most represented of these was the virulence array protein and enolase (the same proteins present in OMP2).

Of the two, only enolase appeared markedly upregulated under iron-deprived conditions when analysed by qPCR (mean fold change of 6.10E + 02, as calculated by the 2^-ΔΔC^_T_ method).

VapA is responsible for the paracrystalline A-layer, an important virulence factor of *A. salmonicida*[4]. Enolase is a virulence factor known to play a role in the plasminogen binding of a number of bacteria, including *Aeromonas hydrophila*[38]. It is also known that the expression of the enolase of *A. hydrophila* is increased during the infection process [39].

No differences were found in the transcription levels of either VapA or enolase between the virulent and avirulent isolates. This finding was consistent with that of Vanden Bergh et al. [40] that determined that the secretion of both of these proteins was not limited in a T3SS deficient mutant.

Band WCP2 contained several proteins of approximately 22kD in size. The most interesting among these was YP_001142399.1, an alkyl hydroperoxide reductase C (AHPC). On the SDS-PAGE, this protein appeared to be more highly expressed in the two virulent isolates under iron-deprived conditions. This was consistent with the results from the RT-qPCR analysis (mean fold change: 5.8E + 01 between virulent and avirulent isolates). To a lesser extent, the transcription of this protein was also upregulated under iron-deprived conditions (3.14E + 01, ranging from 2.23E + 01 to 4.64E + 01). AHPC binds heme molecules and plays a role in its metabolism [41]. However, AHPC has mostly been studied in relation with its role in the resistance against oxidative stress including resistance against macrophages [42]. It has already been demonstrated that APHC is over-expressed in other bacterial pathogens, such as *Vibrio salmonicida* during the infection process [43]. Furthermore, this regulation is sometimes achieved through ferric metabolism [44-46], as seems to be the case for *A. salmonicida*[47].

A fraction of 19 kD in size (WCP3) contained AtpF, a protein known to play a role in the resistance of microorganisms against acidic conditions for example, in *Helicobacter pylori*[48]. SDS-PAGE screening displayed a difference in the intensity of this 19 kD band in the avirulent isolate. This change could be due to one of the proteins within this fraction being down-regulated under iron-deprived conditions. However, when RT-qPCR analysis was conducted to confirm this observation, it failed to detect such downregulation of the transcription levels when the bacterium was cultivated under iron-deprived conditions. Rather, the results indicated that the transcription of the *atpf* gene was slightly upregulated (transcription ratio of 2.50E + 01, ranging from 1.32E + 01 to 4.73E + 01). This suggested that another protein of within this band might have been responsible for the change observed in the band intensity or that this protein’s expression is regulated post-transcriptionally.

Perhaps more interesting was the finding of the H-NS regulator (YP_001142759.1) within the 15 kD band (WCP4). This protein appeared down-regulated under iron-deprived conditions (1.89E + 03, with a confidence interval ranging from 1.09E + 03 to 3.29E + 03). *hns* is part of a multifactor regulatory system in *Shigella flexneri* where it represses transcription of the virulence protein IcsA [49]. *hns* is similarly negatively regulated in the presence of Fur in *Salmonella enterica* serovar *typhimurium*[50] and down-regulation of *hns* in turn allows for the expression of *hil*, resulting in the eventual activation of the *Salmonella* pathogenicity island I (SPI-1). Interestingly, no direct homolog of *hilA* appears present within the genomes of aeromonads. Therefore, if H-NS plays the role of a virulence activator in *A. salmonicida* like it does in the case of *Salmonella* consistent with our findings, it does so through different means.

Contrary to our expectations, only one band from the outer-membrane preparation was found to be overexpressed under iron-deprived conditions. The contents of this band were similar to WCP1, containing both VapA and the enzyme enolase. This contrasted with the results reported by Ebanks *et al*. [30] who, while focussing on outer-membrane proteins, identified three iron-regulated outer membrane proteins (IROMP) on the surface of *A. salmonicida*: a ferric siderophore receptor, a colicin receptor homologue (FstC) and a heme receptor protein. The reason for this discrepancy is not known but several factors might explain it. For example, Ebanks *et al*. have previously suggested that the number of IROMPs might vary depending of the isolate studied [30]. Alternatively, this discrepancy may be related to the method of our preliminary screening. Because we only targeted proteins that appeared differentially expressed on the SDS-PAGE, it is likely that a number of products escaped our detection: Either the difference in their expression levels was too small to be noticeable on the SDS-PAGE, or they were part of a larger fraction and their difference in expression was masked by other proteins in this faction whose expression was constant. This is particularly true for higher molecular weight bands that have poor resolution on 1-D gels. In contrast, Ebanks *et al*. used 2D-gel electrophoresis to isolate the various proteins. Addition of this second dimension yields better resolution of the various proteins thus offering improved sensitivity than the one employed in the present study and these authors were therefore likely to identify proteins that would remain masked using SDS-PAGE, especially higher molecular weight molecules such as the ones, ranging from 73 to 85 kDa, described by these authors [30].

## Methods

### Bacterial strains and culture conditions

Three different isolates of *Aeromonas salmonicida* spp. *salmonicida* were used: strain ATCC 33658 T known to be avirulent [8] and to lack the plasmid carrying the type III secretion system [14,51] and two virulent isolates obtained from disease outbreaks in trout farms in Austria (isolates A-14390 and A-15233). In previous challenges, the strain ATCC 33658 T was shown to induce 70% mortalities when 10^8^ CFU were injected intra-peritoneally, while 10^7^ CFU failed to induced any mortality. In contrast injection of 5x10^3^ CFU induced 90% mortalities when using the isolate A-14390 and 70% mortality with the isolate A-15233). All isolates have been conserved at −80°C and have been used in several fish trials at our institution where they displayed high virulence.

All three isolates were cultured in the same fashion: beads were removed from the −80°C freezer and used to inoculate blood agar plates. After 2 days of cultivation at 15°C, single colonies were removed from these plates and used to inoculate 5 ml of BHI broth with or without iron (iron depletion attained by the addition of 100 μM of the chelator 2’2-dipyridyl to the BHI broth [28]). Cultures were incubated at 15°C for 48 hours before being processed for whole cell crude extract or outer-membrane protein extraction.

### Preparation of the whole cells and outer-membrane proteins

The whole cell crude protein extract was obtained by centrifugation of the bacterial cultures at 3,220 g for 20 min using an Eppendorf 5810R tabletop centrifuge (Eppendorf; Vienna; Austria). Pellets were rinsed 3 times in 0.9 M PBS before being resuspended in 400 μl lysis buffer (50 mM Tris–HCl; 150 mM sodium chloride; 20 mM ethylene diamine tetraacetic acid; 1% sodium deoxycholate; 1% Triton X-100; all ingredients from Sigma; Steinheim am Albuch; Germany). Pellets were sonicated twice on ice for 30 seconds using a UW1070 Sonopuls (Bamdelin; Berlin; Germany). The samples were then centrifuged at 3,220 g at 4°C for 10 minutes and the supernatants were removed and mixed with an equal quantity of Laemmli sample buffer (Bio-Rad; Vienna, Austria).

Outer-membrane proteins (OMPs) were purified based on the protocol by Wilcox [52]. Briefly, the bacterial cultures were pelleted by centrifugation at 3,220 g for 20 minutes and the pellets were rinsed 3 times in 0.9 M PBS before resuspension in 5 ml of 50 mM Tris–HCl and sonication on ice. After centrifugation, supernatants were transferred to a second tube and 500 μl of 20% sodium N-lauroylsarkosynate (Fluka Analytical; Sigma Aldrich; Steinheim am Albuch; Germany) was added. These preparations were then incubated for 30 minutes at room temperature before being ultra-centrifuged at 50,000 g for 1 hour at 4°C in a Sorvall Ultra Pro 80 centrifuge (Thermo-Sorvall; Waltham; USA). Supernatants were set aside and the pellets were resuspended in 15 μl 50 mM Tris–HCl and mixed with 150 μl double-strength Laemmli sample buffer.

### Initial screening and mass spectrophotometry

All preparations were stored at −20°C for no more than 24 hrs. The concentrations within these preparations were then measured by absorbance using a Biophotometer (Eppendorf; Vienna; Austria). Subsequently, the preparations were diluted to the same concentration and an equal amount of total protein were resolved by SDS-PAGE using a 4% polyacrylamide stacking gel and a 16% resolving gel [53].

Gels were run using the Mini-Protein Tetra Cell (Bio-Rad laboratories GmbH) at 120 V for 70 min in 1× running buffer (25 mM Tris, 250 mM glycine, pH 8.3, 0.1% SDS). Proteins were visualized by staining with Coomassie Brilliant Blue R-250 (Sigma-Aldrich) and the Precision plus protein all blue standard (Bio-Rad, Vienna, Austria) was used to estimate the molecular weight of the separated proteins.

Bands that appeared differentially expressed, either between the virulent isolates and the avirulent strain or under iron rich and iron-deprived culture conditions, were excised. These bands were subsequently digested *in*-*gel* using trypsin (Trypsin Gold, Mass Spectrometry Grade, Promega, Madison, WI, USA). The extracted peptides were then analyzed in a nano-LC MS/MS using an Ultimate 3000 RSLC HPLC system (Dionex, Amsterdam, Netherlands) followed by mass spectrometric analysis in a HCT Esquire mass spectrometer (Bruker Daltonics, Bremen, Germany). Processed spectra were identified using Mascot in the NCBI database (Table 1). In order to identify the proteins that were likely to be the most important contributor to the detected variations, we employed the peptide-matching score summation (PMSS) technique which scores the various proteins depending on their relative abundance.

### Design of the oligonucleotide primers and preparation of the standard curves

Based on the initial screening and mass spectrometry results, five genes of particular interest were selected for investigation by quantitative RT-qPCR: *alkyl hydroperoxide reductase C* (*ahpC*), ATP-*synthetase F* (ATP-F), *enolase*, *histone*-*like nucleoid structuring protein* (*hns*) and the *virulence array protein* (*vapa*). Several proteins identified in the gels were known to be housekeeping genes or genes unlikely to be involved in virulence and were therefore not included further in the study. Primers were designed using the software Primer 3 to target short sequences (ranging from 112 bp to 180 bp in size) within these genes as well as within the 16S RNA sequence (Table 3). The optimum annealing temperature for each primer was determined using a gradient PCR assay and standard curves were generated for its gene using ten-fold dilutions (from 10^−1^ to 10^−10^).

**Table 3:** List of the primers used in this study

**Primer Name**	**Sequence**	**Gene targeted**	**Amplicon size**
FdAS-16SRNART	ATATTGCACAATGGGGGAAA	16S RNA	148 bp
RvAS-16SRNART	GTTAGCCGGTGCTTCTTCTG		
FdAS-AHPCRT	CCTACCACAATGGCAAGTT	Alkyl Hydroperoxide Reductase C	180 bp
RvAS-AHPCRT	TCGGTGGAGACGGAGTAGA		
FdAS-AtpF RT	CGGTCAGACACTTGCTTTCA	*atpF* gene product	149 bp
RvAS-AtpFRT	TGGCCAAATCCAGATCTTTC		
FdAs-EnoRT	GATACGACCTTCGCCTTTCA	Enolase	112 bp
RvAs-EnoRT	TGCTGCTGGCTATGTACTGG		
FdAS-H-NS-RT	CGTTTGAACAGCTGCAAGAA	*hns* Transcriptional regulator	127 bp
RvAS-H-NS-sRT	ATTTCGGTGAACTCGCTCAG		
FsAS-VapART	TGTTCCGGTTCCTGTTGTT	Virulence array protein (VapA)	177 bp
RvAS-VapART	CACGAATGTGGATGAGGTTG		

### RNA isolation and cDNA synthesis

*A. salmonicida* spp. *salmonicida* were grown in triplicate at 15°C under the conditions previously described. Subsequently, bacterial cells were lysed in lysis buffer and homogenised using a QIAshredder kit (Qiagen, Hilden, Germany). The RNA was isolated from these lysates using a Qiagen RNeasy kit (Qiagen, Hilden, Germany) according to the manufacturer’s instructions. The samples were treated *on*-*column* with DNAse (Qiagen, Hilden, Germany) to exclude DNA contamination and eluted in 20 μl RNAse free water. cDNA was synthesised using the iScript cDNA synthesis kit (Bio-Rad, Vienna, Austria) following the manufacturer’s instructions.

### Quantitative real-time PCR assay

Real-Time PCR (qPCR) was carried out in 25 μl end-volumes in technical duplicates using a CFX96 Touch real-time PCR detection system (Bio-Rad, Vienna, Austria). The reaction mixtures comprised of 5 μl cDNA template, 12.5 μl SYBR Green Supermix dye (Bio-Rad, Vienna, Austria) including a 7 pmol final concentration of each primer. The qPCR program started with 3 minutes at 95°C, followed by 39 cycles of 94°C, 59°C and 72°C, at 30 seconds each, with the plate reading step at 59°C. The thermal profile for the melt-curve analysis was determined by incubation at 59°C for 1 minute followed by gradual increase of temperature to 95°C during which change in fluorescence was monitored. No template controls (NTCs) were included in duplicate to control for the presence of primer-dimers. The results obtained were analysed using the Bio-Rad CFX Manager 2.1 software (Bio-Rad, Vienna, Austria). The ΔCT values obtained were normalized based on the 16S-RNA and the normalized transcription ratios were calculated based on the 2^-ΔΔC^ method [54] in order to compare the gene transcription levels between the virulent and avirulent strains and also within strains between the two culture conditions.

## Conclusions

In the present study, we used SDS-PAGE, mass spectrometry and RT-qPCR to analyze the protein expression profiles of three strains of *A. salmonicida* ssp. *salmonicida* under normal and iron deprived culture conditions. We determined that the protein AHPC which is involved in resistance against oxidative stress was over-expressed in the virulent isolates compared to the avirulent isolates including marginal over-expression under iron poor conditions in all isolates. Similarly, the enzyme enolase was overexpressed when the bacteria were cultivated in broth enriched with 2’2 dipyridyl. In contrast, the regulatory protein H-NS was under-expressed under such iron-limited condition which suggests the existence of a regulatory pathway coordinated through this molecule. This putative pathway however, does not act through *hilA* as is the case in the genus *Salmonella* because no homologue of the molecule could be identified in the aeromonads’ genome. Thus we believe that these findings enhance our understanding of the virulence mechanisms and regulation in *A. salmonicida*. However, additional research will be required to ascertain the precise mechanisms by which the availability of iron regulates the expression of the genes identified in this study.

## Abbreviations

AhpC: Alkyl hydroperoxide reductase C; Asap1: *Aeromonas salmonicida* protein 1; ATCC: American Type Culture Collection; ATPF: ATP-synthetase F; BHI: Brain Heart Infusion; DapD: Diaminopimelate biosynthesis D; Fap: Fibril assembly protein; Frr: Ribosome recycling factor; Flp: Fibril-associated protein; Fur: Ferric uptake regulator; H-NS: Histone-like nucleoid structuring protein; HCl: Hydrogen Chloride; HCT: High capacity ion trap mass spectrometer; HilA: Hyperinvasive locus A; HPLC: High-performance liquid chromatography; HutE: Histidine utilizing Protein E; MS: Mass Spectrometry; OMP: Outer-Membrane Protein; PBS: Phosphate Buffered Saline; PMSS: Peptide-matching score summation technique; NCBI: National Center for Biotechnology Information; NTC: No template controls; qPCR: Quantitative real-time PCR; RPLE: Ribosomal Protein LE; RPLF: Ribosomal Protein LF; RT-qPCR: Reverse transcription quantitative real-time PCR; SDS-PAGE: Sodium Dodecyl Sulfate -Polyacrylamide gel electrophoresis; T3SS: Type 3 Secretion System; VapA: Virulence array protein; WCP: Whole-cell protein.

## Competing interests

The authors declare that they have no competing interests.

## Authors’ contribution

SML planned the study, designed the primer and drafted the manuscript. In addition SML, alongside JK, performed the SDS-PAGE and RT-qPCR. KN extracted the peptides and performed the analysis by mass-spectrophotometry and identification by Mascot. MEL supervised the study and helped with the revision of the draft. All authors read and approved the final manuscript.

## Supplementary Material

Additional file 1: Figure S1 SDS-PAGE of the outer-membrane preparation displaying OMP2. Well A contains Isolate A-15233 (virulent) cultivated under normal conditions while well B contains Isolate A-15233 (virulent) cultivated under iron-deprived. Click here for file
