# Supplementary figures and images for: Protein expression and transcription profiles of three strains of Aeromonas salmonicida ssp. salmonicida under normal and iron-limited culture conditions
Source: Proteome Sci. 2014 May 19;12:29. doi: 10.1186/1477-5956-12-29 (PMC4035829; doi:10.1186/1477-5956-12-29)

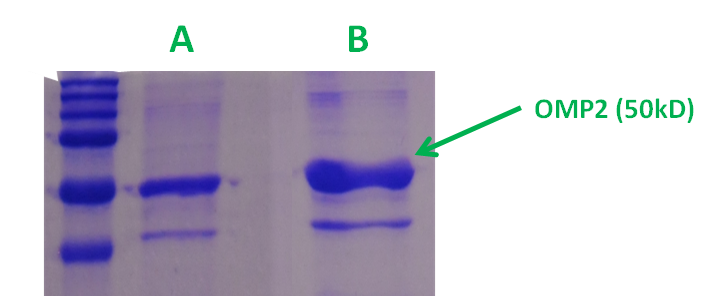

Supplement: Additional file 1: Figure S1 — SDS-PAGE of the outer-membrane preparation displaying OMP2. Well A contains Isolate A-15233 (virulent) cultivated under normal conditions while well B contains Isolate A-15233 (virulent) cultivated under iron-deprived. [file 1477-5956-12-29-S1.png]
